# The effect of time on task, sleep deprivation, and time of day on simulated driving performance

**DOI:** 10.1093/sleep/zsac167

**Published:** 2022-07-22

**Authors:** Isabella Marando, Raymond W Matthews, Linda Grosser, Crystal Yates, Siobhan Banks

**Affiliations:** Behaviour-Brain-Body Research Centre, Justice and Society, University of South Australia, Adelaide, SA, Australia; Human Performance and Safety, Royal Australia Air Force, Adelaide, SA, Australia; Behaviour-Brain-Body Research Centre, Justice and Society, University of South Australia, Adelaide, SA, Australia; Behaviour-Brain-Body Research Centre, Justice and Society, University of South Australia, Adelaide, SA, Australia; Behaviour-Brain-Body Research Centre, Justice and Society, University of South Australia, Adelaide, SA, Australia

**Keywords:** driving performance, workload, time on task, sleep deprivation, time of day, sustained attention

## Abstract

Sleep deprivation and time of day have been shown to play a critical role in decreasing ability to sustain attention, such as when driving long distances. However, a gap in the literature exists regarding external factors, such as workload. One way to examine workload is via modulating time on task. This study investigated the combined effect of sleep deprivation, time of day, and time on task as a workload factor on driving performance. Twenty-one participants (18–34 years, 10 females) underwent 62 h of sleep deprivation within a controlled laboratory environment. Participants received an 8-h baseline and 9.5-h recovery sleep. Every 8 h, participants completed a Psychomotor Vigilance Task (PVT), Karolinska Sleepiness Scale (KSS), 30-min monotonous driving task and NASA-Task Load Index (TLX). Driving variables examined were lane deviation, number of crashes, speed deviation and time outside the safe zone. Workload was measured by comparing two 15-min loops of the driving track. A mixed model ANOVA revealed significant main effects of day and time of day on all driving performance measures (*p* < .001). There was a significant main effect of workload on lane deviation (*p* < .05), indicating that a longer time on task resulted in greater lane deviation. A significant main effect of day (*p* < .001) but not time of day for the NASA-TLX, PVT and KSS was found. Time on task has a significant further impact on driving performance and should be considered alongside sleep deprivation and time of day when implementing strategies for long-distance driving.

**Statement of Significance**

The amount of sleep and time of day impair cognitive performance, especially sustained attention. However, little research has specifically examined the role of workload on long sustained attention tasks, such as driving performance. Results showed that workload (time on task) influenced lane deviation, indicating that the longer the participants drove, the more their performance deteriorated. Higher subjective workload scores also tracked poorer driving performance. Future research is required to investigate the impact of other workload factors, such as cognitive load on driving performance, to encapsulate the whole construct.

## Introduction

Sleep loss and time of day, impact sustained attention, and performance [1–4]. These factors are one of the major contributors to road accidents globally, with fatigue accounting for 20% of motor-vehicle crashes [5]. Numerous studies have found that sleep deprivation has a negative effect on driving performance similar to that of alcohol [6, 7]. Research has also found there is an eleven-fold increase in the risk of having a fatal motor crash whilst driving on a highway at 04:00, compared to during the day [8].

Alongside these processes, external factors, including workload, impact alertness, and reduce cognitive performance [9]. There is debate surrounding how workload is conceptualized and operationalized, but it is commonly defined as how demanding the task is regarding time on task and expended efforts to meet demands [9, 10]. When workload is too high, alertness decreases due to an inability to cope with demands [11]. Conversely, if workload is too low then errors may also appear due to boredom and loss of sustained attention [12]. Workload is a complex construct, as there are various factors that impact workload and influence the maintenance of alertness.

Time on task is a primary factor contributing to workload, as extended time on a task can increase sleepiness and reduce alertness [13]. This may be due to areas of the brain “falling asleep” when over worked, even while the individual, is functionally awake. This has been demonstrated in rodent studies, where rat’s whiskers were twitched repeatedly, to stimulate the rat, whilst brain activity was monitored with electroencephalogram (EEG) [13]. The overworked area of the rodent’s brain displayed characteristics of “local sleep”, suggesting that the area of the brain had gone to sleep. This suggests that with extended wakefulness or sustained engagement with a task, the neural circuits which support attention can become exhausted and shut down. While an individual is otherwise functionally awake, these brain changes could lead to performance instability leading to reduced sustained attention and an increase in errors.

Driving requires continuous sustained attention [14]. Monotonous driving can be characterized by repetitive, mundane, straight roads where there is little traffic and high predictability, such as highway driving [15]. Although the task demands regarding cognitive load and information processing are low, monotonous driving can have a demanding workload due to the effort required to sustain attention [16–18]. The effect of monotonous driving was studied using a 40-min driving simulator task during the post-lunch circadian dip (13:30–15:00) [19]. Researchers found that performance significantly decreased across the task, with major increases in lane deviation beginning from 20 min into the drive, indicating that inattentiveness manifests rapidly during these mundane driving conditions.

Along with performance measures of alertness and workload, it is important to concurrently measure subjective perceptions as they help predict behavior [20], and prompt the use of countermeasures, such as taking a break during a long drive. Subjective workload perceptions are a useful tool as they can identify small changes in workload that may be otherwise difficult to detect [10]. Subjective measures are also valid indicators of changes to mental workload [20], and alertness [21]. Taking the above factors into consideration, it is imperative that both objective performance and subjective measures are used in tandem to examine alertness and workload.

The literature shows that sleep deprivation, time of day, and time on task all individually impact sustained attention. However, little is known about how time on task as a workload factor impacts sustained attention, specifically driving performance, at different times of the day under conditions of sleep deprivation. It is common within Australia for long-haul truck drivers to operate a vehicle between 6 and 14 h with little or no breaks [22, 23]. This shows the importance that understanding workload in the context of driving, whilst sleep deprived and at different times of the day. Therefore, the aim of this study was to investigate the impact of two nights of sleep deprivation, various times of day and time on task on driving performance.

## Methods

### Participants

A priori power analysis was conducted to compute the required sample size using G*Power 3.1 [24]. The specific approach taken on G*Power was for an ANOVA: repeated measures, within factors. The analysis indicated that a sample size of 8 would be required for adequate power of 0.8 at a significance alpha of 0.05 [25]. The effect size of 0.84 was obtained from a study investigating the effect that one night of sleep deprivation had on driving performance [26]. The previous study explored driving performance throughout a 40-min drive, however the values at the 30-min mark of the baseline and post-shift drives were used in the no-nap condition for a within-groups comparison. Due to the longer period of sleep deprivation in the current study, the required sample size was increased to account for possible participant attrition.

A total of 23 healthy adults (11 females and 12 males), aged 18–34 years old, were recruited to participate in this study. Exclusion criteria consisted of individuals with poor sleep patterns, excessive caffeine use, concerning health, or psychiatric disorders, regular/heavy drug, or alcohol use, and the use of any corticosteroid or anti-inflammatory medications. Refer to Table 1 for the screening questionnaires utilized, along with their justification and exclusion criteria.

**Table 1.** Questionnaires for screening with justification and exclusion criteria

Screening Questionnaires	Justification	Exclusion criteria
Demographic Information Questionnaire	To identify demographic information which can be useful in generalizing results beyond the study	Reported shift work within the past month, trans-meridian travel within the past 2 months, significant medical events, medication, or substance use, excessive alcohol or caffeine use and smoking
Pittsburgh Sleep Quality Index	Assesses sleep quality over the past month to identify whether participants meet inclusion and exclusion criteria for sleep	Scores > 5 indicating sleep difficulties
Sleep/ Wake Survey	To understand sleeping patterns to ensure that participants meet the inclusion and exclusion criteria for sleep	Reported negative experiences with sleep loss, *trans*-meridian travel, significantly early/ late bedtime/wakeup time, habitual napping and excessive alcohol, caffeine or drugs use
Composite Morningness and Eveningness Scale	To understand participants natural circadian rhythms and identify if they are a morning or evening type	Scores ≤ 22 indicating evening type or scores ≥ 44 indicating morning type
Confidential Medical Screen	To ensure participants are healthy to allow for generalisability of results	Any significant health issues, use of medication or illicit drugs
Beck Depression Index	To identify whether participants are mentally healthy and not experiencing any symptoms of depression	Scores > 14 indicating mild mood disturbance OR if responded c or d to question 9 (regarding suicidality)
Berlin Questionnaire	To identify whether participants are at risk of sleep apnea	If ≥ 2 categories are scored positively, indicating high risk of sleep apnea
Stop Bang Questionnaire	To identify whether participants are at risk of sleep apnea	If ≥ 2 questions are answered “yes”, indicating risk of sleep apnea
Handedness Questionnaire	To identify the participants preferred hand, as some tasks need to be accommodated to left handers	N/A

Recruitment occurred through posters placed on notice boards throughout the University of South Australia (UniSA). Eligible respondents were contacted for further screening procedures. At the completion of the study, participants received an honorarium of $600. Ethical approval was obtained from the UniSA Human Research Committee (Application ID: 202112).

### Design

**Figure 1.**
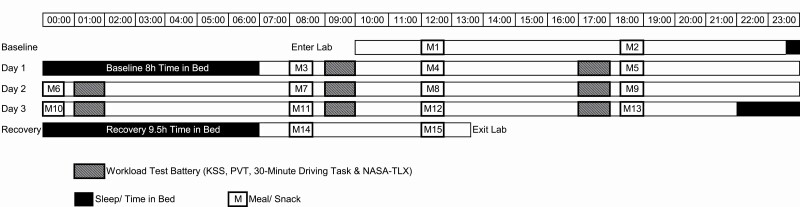
Protocol Diagram. Participants entered the laboratory at 10:00. Workload test batteries are denoted by striped bars, which included the Karolinska Sleepiness Scale (KSS), Psychomotor Vigilance Task (PVT), 30-minute driving task and NASA-Task Load Index (TLX). Sleep/Time in Bed is demonstrated by black bars, meals are illustrated by white bars. Participants exited the lab at 13:30.

A within-groups, repeated measures experimental design (Figure 1) was employed to explore the combined effect that sleep deprivation, time of day and time on task has on sustained attention.

### Materials and measures

#### Location

The experiment was conducted in the Sleep and Chronobiology Laboratory at UniSA. The highly controlled laboratory has a constant temperature of 22°C, light fixed at 100 lux, no windows, and is soundproof, to ensure no exogenous variables misalign natural circadian rhythms.

#### Materials for screening procedures

Interested respondents were initially screened via a telephone interview whereby relevant medical, sleep, and lifestyle disorders were discussed. If deemed eligible, participants visited the Sleep and Chronobiology Laboratory where the aims and expectations of the study were firstly explained, and signed consent was obtained. Participants were then asked to complete screening questionnaires pertaining to sleep quality, morning, or evening preference, depression levels, sleep apnea symptoms and handedness preference.

A urine sample was taken to screen for drugs and a full blood test done for general health physiological screening, with specific analyses looking at electrolytes, thyroid stimulating hormone and liver functioning. One week prior to the experiment, participants completed a sleep-wake diary and wore a GENEActive [27] monitor, to ensure a minimum of 7 h sleep per night was obtained, with sleep onset before midnight and wake before 09:00.

#### Driving task

A driving simulation was employed to measure sustained attention and the effect of time on task, as a workload factor [28].

**Figure 2.**
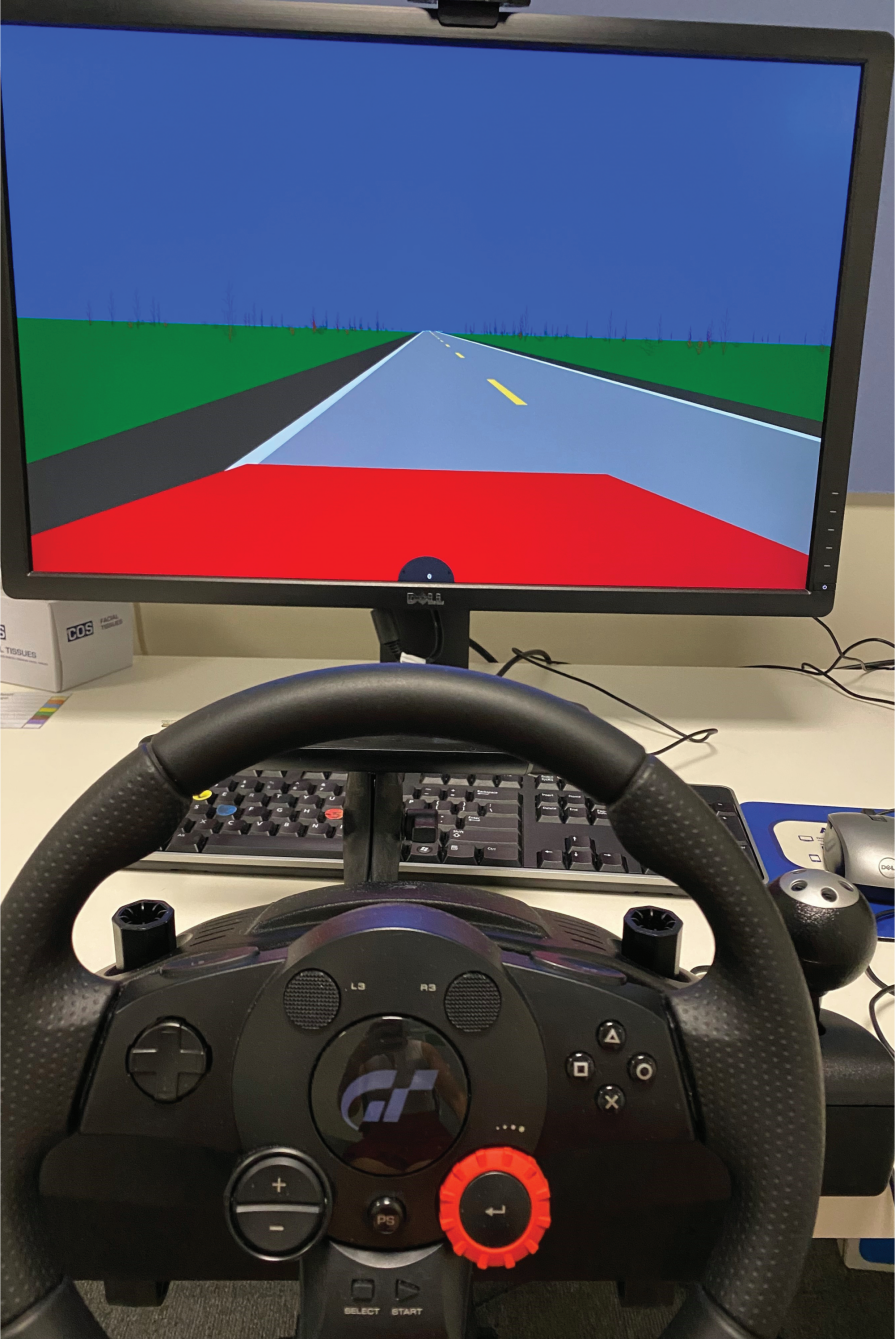
Driving task setup.

A customized 30-min track was developed by York Computer Technologies to measure the effect that time on task has on sustained attention. To examine time on task as a workload factor, two 15-min loops were examined, with the second loop having a cumulative longer time on task. Each loop around the driving track took 15-min (when adhering to speed and driving instructions), making the two loops directly comparable. The track mimicked a monotonous country highway drive, with a mundane scenery of shrubs (Figure 2). There was one lane traveling in each direction, with four oncoming vehicles passing per loop.

**Figure 3.**
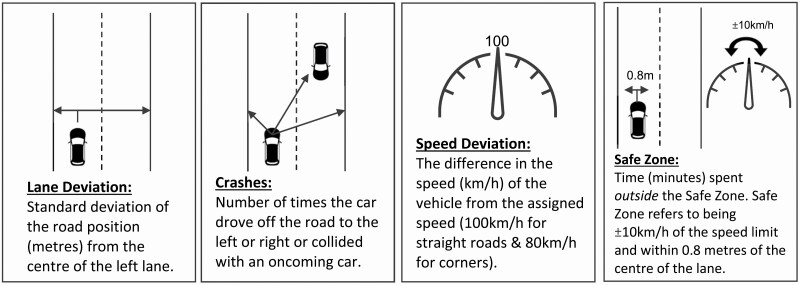
Variables used for analysis from the driving task. Adapted from “It’s not just what you eat but when: The impact of eating a meal during simulated shift work on driving performance”, by C. C. Gupta et al. [29], *Chronobiology International, 34*(1), p. 69 (https://doi.org/10.1080/07420528.2016.1237520). Copyright 2020 by Informa UK Limited.

A steering wheel, accelerator, and brake were installed on the computers in participants bedrooms, to simulate a real driving experience. Participants were instructed to keep both hands at the 10 and 2 position for the duration of the drive, adhere to speed limits (100 km/h on straight roads and 80 km/h at corners), and stay in the center of the left lane. If participants crashed, there was a crash sound followed by instructions for them to promptly restart the drive. The primary outcome variables of this study, derived from this task, were lane deviation, crashes, speed deviation, and safe zone (Figure 3).

#### Subjective workload—NASA-Task Load Index (TLX)

The NASA-Task Load Index (TLX) was utilized to measure the perception of workload demands [30]. The NASA-TLX is the gold-standard measure for perceived mental workload [31]. This scale contains six questions, outlining the six main factors contributing to workload, being mental demand, physical demand, temporal demand, performance, effort, and frustration [11]. Each question has a maximum score of 100, with a higher score indicating a more demanding workload. The NASA-TLX was administered at the conclusion of the workload tests to ascertain the experienced workload levels. Within the current sample, high internal consistency was established amongst the subscales of the NASA-TLX, *a* = .838. The variables utilized for analysis were the scores from the six subscales, along with the overall average score [30].

#### Behavioral alertness—Psychomotor Vigilance Task (PVT)

The PVT was utilized to measure the effect of sleep deprivation and time of day on alertness. The PVT is the current gold-standard measure of alertness and psychomotor vigilance with minor learning and aptitude effects [32].

The 10-min PVT occurred after the driving task. Participants were instructed to respond as quickly as possible to the red stimulus by pressing the button and were warned against false starts (i.e. responding before stimulus appeared). During the task, the reaction time in milliseconds was displayed for 1 s, to serve as performance feedback. The PVT has variable inter-stimulus intervals, between 2 and 10 s, yielding approximately 90 stimuli per trial [33]. The main PVT outcome variable is the reciprocal reaction time (RRT; 1/Reaction Time × 1000), which measures the average response speed [34].

#### Subjective alertness—Karolinska Sleepiness Scale (KSS)

The Karolinska Sleepiness Scale (KSS) was completed at the beginning of every workload test battery as a subjective measure of situational alertness [35]. The KSS is a one-dimensional, 9-point self-reported scale ranging from *“extremely alert”* to *“extremely sleepy; fighting sleep”*.

### Procedure

The protocol consisted of a baseline day, three experimental days, and a recovery day (Figure 1). On the baseline day, participants practiced the test batteries, to ensure familiarity and received an 8-h baseline sleep (23:00–07:00).

The following day participants began the 62 h of sleep deprivation. The rationale for this amount of sleep deprivation was that it allowed for at least two full 24-h circadian cycles of data collection. This was also to account for the vast inter-individual differences in performance that are presented under conditions of sleep deprivation [36]. During this time, participants completed eight workload test batteries, occurring every 8 h, at 09:00, and 17:00 on day 1 and at 01:00, 09:00, and 17:00 on days 2 and 3. Baseline and recovery sleep was measured using polysomnography. The workload test batteries consisted of a KSS, driving task, PVT, and the NASA-TLX.

When participants were not completing cognitive tasks, they were encouraged to congregate in the living area to engage in non-strenuous activities, such as watching movies. To maintain control, participants received meals based on calorie and macronutrient requirements as calculated by a nutritionist. Participants were not allowed caffeine or other stimulants. After 62 h of sleep deprivation, participants received a 9.5-h recovery sleep opportunity.

### Statistical analysis

**Figure 4.**
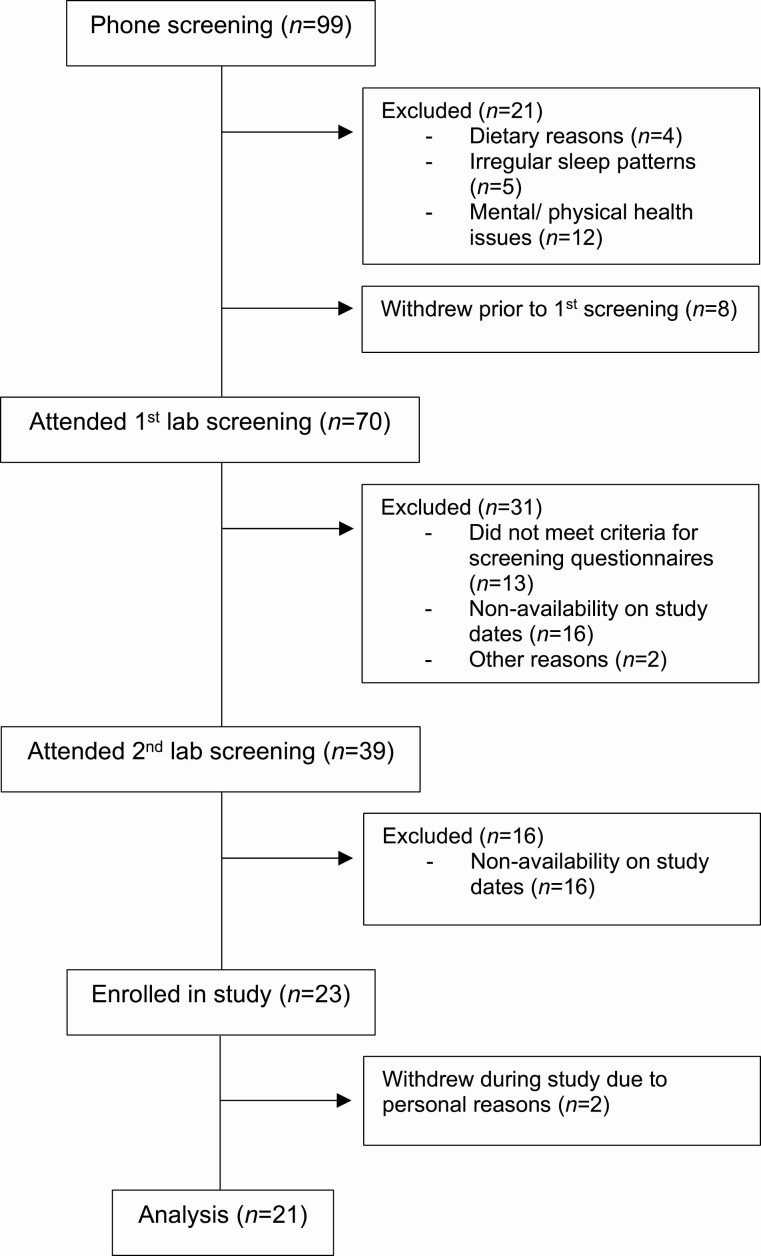
CONSORT diagram of participants.

Statistical analyses were conducted on SPSS [37]. Two participants left the study early for personal reasons; thus, all their data was excluded from analyses (Figure 4). After the exploration of descriptive statistics, a linear mixed model ANOVA was conducted. To explore the effect that sleep deprivation and time of day had on alertness, the fixed factors of day (1–3), and time of day (09:00, 17:00 and 01:00) were used for PVT RRT and KSS scores. Overall average on the NASA-TLX and each NASA-TLX subscale were assessed with the fixed factors of day and time of day to explore subjective workload. The NASA-TLX performance subscale was reverse scored for clear comparisons; therefore, higher scores indicate poorer performance. To explore time on task as a workload factor, the 30-min drive was split into two comparable 15-min loops. The variables of lane deviation and safe zone were calculated with crashes omitted, so only deviations which did not result in an off-road collision were included. The fixed factors of day, time of day, and time on task (loops 1 and 2) were used to assess lane deviation, crashes, speed deviation, and safe zone. For all mixed models “Participant ID” was entered as a random effect on the intercept to account for between-subject variability. The assumption that the residuals of the model were normally distributed was met for all variables. To identify significant differences between day and time of day, pairwise comparisons were explored using the Šidák [38] correction. Significance was acknowledged at a threshold of *p* < .05. Effect sizes were calculated using Partial Eta Squared. In accordance with Richardson [39], small, medium, and large effects were determined with the thresholds of 0.01, 0.06, and 0.14, respectively. PSG recordings were scored by a sleep technician and the average total sleep time in minutes for baseline and recovery were obtained. A reliability analysis was conducted on the NASA-TLX subscales to measure internal consistency.

## Results

The final sample consisted of 21 healthy participants (Table 2). Descriptive statistics are presented in Table 3. For full inferential results and effect sizes, refer to Table 4.

**Table 2.** Demographic characteristics of the overall sample

Demographic information	All participants *N* = 21
Age in years Mdn (range)	23 (18–34)
Gender *N* (%)	
Female	10 (47.6)
Male	11 (52.4)
BMI^1^	21.9 (3.2)
CMS^1^	36 (4.4)
PSQI^1^	4 (2.4)
TST—Baseline sleep^1^	439.6 (15.9)
TST—Recovery sleep^1^	552.8 (9.3)

^1^ *M*(*SD*); *BMI* Body Mass Index; *CMS* Composite Morningness and Eveningness Scale, evening types (≤ 22) and morning types (≥ 44) were excluded from the study; *PSQI* Pittsburgh Sleep Quality Index, participants were excluded if PSQI score was > 5; *TST* total sleep time (min).

**Table 3.** Means and standard deviations for all variables across the study

	Day 1				Day 2						Day 3					
	09:00		17:00		01:00		09:00		17:00		01:00		09:00		17:00	
	*M*	*SD*	*M*	*SD*	*M*	*SD*	*M*	*SD*	*M*	*SD*	*M*	*SD*	*M*	*SD*	*M*	*SD*
PVT RRT	3.95	0.333	3.80	0.490	3.422	0.562	3.15	0.460	3.35	0.394	2.91	0.689	2.59	0.499	3.07	0.604
KSS	3.10	1.09	4.24	1.64	5.57	1.89	6.24	1.73	5.95	1.50	6.76	1.84	7.62	1.32	5.95	1.66
NASA-TLX																
Overall	28.49	11.16	31.00	13.81	35.02	13.58	49.39	11.32	36.23	14.13	42.81	14.28	47.41	15.00	41.07	13.81
Mental	30.75	31.22	28.44	30.03	29.84	30.19	55.03	35.30	35.70	31.19	42.05	37.61	45.42	36.61	41.81	31.99
Physical	15.62	21.39	19.47	23.83	22.56	23.07	39.44	35.43	26.35	28.10	32.95	31.09	36.29	34.76	33.35	30.40
Temporal	20.40	21.06	18.97	22.16	25.61	23.50	34.46	27.65	27.25	24.49	29.01	26.38	32.77	29.87	30.71	25.32
Performance	42.27	31.95	55.26	29.76	57.17	30.20	66.17	24.71	63.26	29.97	69.79	28.22	75.30	24.80	65.35	25.71
Effort	34.87	31.64	38.23	32.9	46.09	31.04	49.47	28.86	37.87	28.26	41.74	32.95	49.07	34.25	46.91	32.21
Frustration	23.02	26.39	25.66	30.68	28.83	25.56	51.74	34.89	26.97	29.46	41.29	35.59	45.61	38.11	28.31	32.93
Driving task																
Lane deviation	0.392	0.126	0.413	0.163	0.485	0.224	0.847	0.288	0.673	0.215	0.805	0.252	1.03	0.332	0.840	0.299
Crashes	0.095	0.294	0.238	0.539	1.58	3.42	21.78	29.1	7.48	10.5	19.24	24.81	46.7	44.12	18.2	18.8
Speed deviation	‐2.38	3.08	‐1.43	2.47	‐2.08	4.66	‐6.18	7.58	‐1.69	4.76	‐7.85	7.26	‐11.39	10.35	‐4.67	7.39
Safe zone	6.75	5.06	6.00	4.05	9.35	7.67	15.72	7.9	11.88	8.20	15.20	8.15	19.71	7.75	15.78	9.05

Scores for the NASA-TLX performance subscale were inverted for easier interpretation.

*PVT RRT* psychomotor vigilance task reciprocal reaction time; *KSS* Karolinska Sleepiness Scale; *NASA-TLX* NASA-Task Load Index.

**Table 4.** Results of the linear mixed models ANOVA

	Day		Time of day		Time on task		Day*time of day		Day*time on task		Time of day*time on task		Day*time of day* time on task	
	*F*_*df*_	*η*_*p*_^2^	*F*_*df*_	*η*_*p*_^2^	*F*_*df*_	*η*_*p*_^2^	*F*_*df*_	*η*_*p*_^2^	*F*_*df*_	*η*_*p*_^2^	*F*_*df*_	*η*_*p*_^2^	*F*_*df*_	*η*_*p*_^2^
PVT RRT	**46.91**_**2,154**_ ^**^	0.378	2.66_2,154_	0.033			2.87_3,154_	0.046						
KSS	**46.41**_**2,160**_ ^**^	0.367	0.855_2,160_	0.010			**5.60**_**3,160**_ *	0.095						
NASA-TLX														
Overall	**26.45**_**2,157**_ ^**^	0.252	2.60_2,157_	0.032			1.85_3,157_	0.034						
Mental	**13.65**_**2,157**_ ^**^	0.148	3.04_2,157_	0.037			0.604_3,157_	0.011						
Physical	**16.53**_**2,157**_ ^**^	0.173	1.26_2,157_	0.015			1.09_3,157_	0.020						
Temporal	**11.70**_**2,157**_ ^**^	0.129	1.15_2,157_	0.014			0.311_3,157_	0.005						
Performance	**16.78**_**2,157**_ ^**^	0.176	0.267_2,157_	0.003			2.09_3,157_	0.038						
Effort	**9.54**_**2,157**_ ^**^	0.108	0.575_2,157_	0.007			1.54_3,157_	0.028						
Frustration	**10.24**_**2,157**_ ^**^	0.115	**3.26**_**2,157**_ *	0.039			2.42_3,157_	0.044						
Driving task														
Lane deviation	**106.91**_**2,306**_ ^**^	0.411	**23.40**_**2,306**_ ^**^	0.132	**4.18**_**1,306**_ *	0.013	**4.57**_**3,306**_ *	0.042	0.063_2,306_	0.003	0.508_2,306_	0.003	0.237_3,306_	0.002
Crashes	**47.95**_**2,306**_ ^**^	0.238	**20.81**_**2,306**_ ^**^	0.119	0.124_1,306_	0.000	**5.33**_**3,306**_ *	0.049	0.092_2,306_	0.000	0.159_2,306_	0.001	0.606_3,306_	0.005
Speed deviation	**30.13**_**2,306**_ ^**^	0.164	**9.48**_**2,306**_ *	0.058	0.341_1,306_	0.001	2.32_3,306_	0.022	0.495_2,306_	0.003	0.052_2,306_	0.000	0.104_3,306_	0.001
Safe zone	**56.06**_**2,306**_ ^**^	0.268	**9.35**_**2,306**_ ^**^	0.057	2.10_1,306_	0.006	1.19_3,306_	0.011	0.181_2,306_	0.001	0.237_2,306_	0.001	0.463_3,306_	0.004

Time on task was only measured within the driving task variables. Effect size thresholds are 0.01, 0.06, and 0.14 for a small, medium, or large effect size, respectively Richardson [38].

*F*_*df*_ *F* value with degrees of freedom (displayed in subscript); *η*_*p*_^2^ partial eta^2^; *PVT RRT* psychomotor vigilance task reciprocal reaction time; *KSS* Karolinska Sleepiness Scale; *NASA-TLX* NASA-Task Load Index. Bold values indicate statistical significance.

*p < .05,

^**^ *p* < .001.

### Sustained attention task—driving task

#### Lane deviation

**Figure 5.**
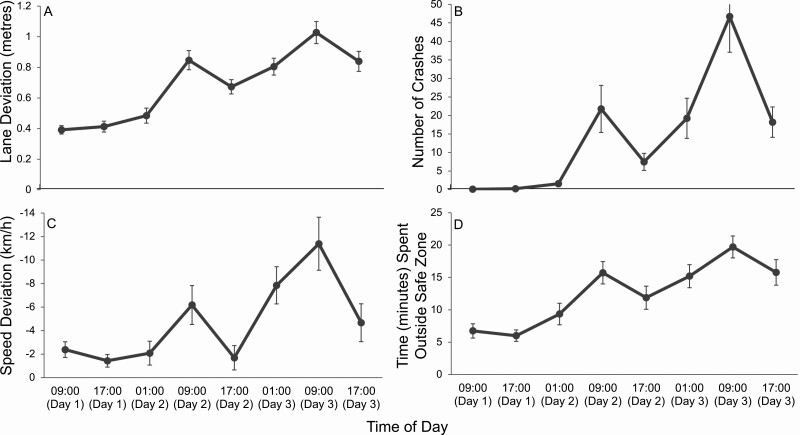
Mean scores during the 30-min driving task, at each time of day. Mean scores across each time of day. Error bars display the standard error. (A) Lane deviation refers to the standard deviation of the road position (m) from the center of the left lane. (B) Crashes occur when the vehicle drove off the road to the left or right or collided with an oncoming car. (C) Speed deviation refers to the difference in speed (km/h) from the assigned speed limit. The speed limit was 100 km/h on straight roads and 80 km/h at corners. Negative numbers indicate driving under the speed limit. (D) Time in minutes spent outside the safe zone. The safe zone refers to being within ± 10 km/h of the sleep limit and within 0.8 m of the center of the lane. Descriptive statistics are presented in table form in Table 3.

**Figure 6.**
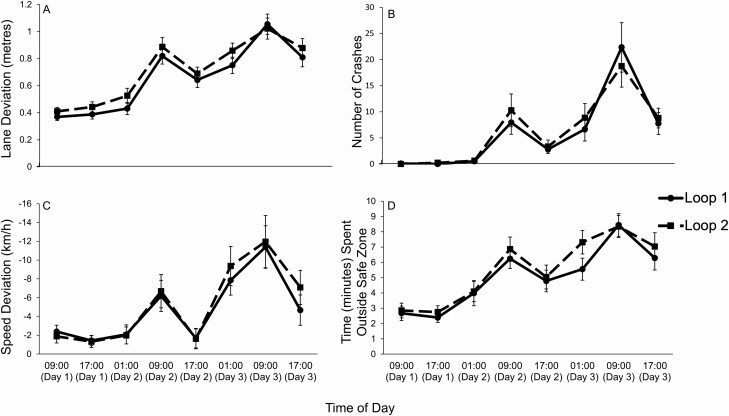
Mean scores of Loop 1 and Loop 2, during the 40-min driving task, at each time of day. Mean scores across each time of day. Error bars display the standard error. Loop 1 is indicated by the full line and Loop 2 is indicated by the dashed line and has a cumulative longer time on task. Each Loop took 15-min to complete. (A) Lane deviation refers to the standard deviation of the road position (m) from the center of the left lane. (B) Crashes occur when the vehicle drove off the road to the left or right or collided with an oncoming car. (C) Speed deviation refers to the difference in speed (km/h) from the assigned speed limit. The speed limit was 100 km/h on straight roads and 80 km/h at corners. Negative numbers indicate driving under the speed limit. (D) Time in minutes spent outside the safe zone. The safe zone refers to being within ± 10 km/h of the sleep limit and within 0.8 m of the center of the lane. Descriptive statistics are presented in table form in Table 3.

Significant main effects of day (*p* < .001), time of day (*p* < .001; Figure 5a), and time on task (*p* = .042; Figure 6a), were found for lane deviation. Pairwise comparisons revealed significant increases in lane deviation with increased sleep deprivation (*p* < .001). Pairwise comparisons revealed that lane deviation differed based on the time of day, with best performance around the circadian peak (17:00), and worst performance around the circadian nadir (09:00). Significant time of day differences were found between 09:00 and 17:00 (*p* < .001), as well as 09:00 and 01:00 (*p* = .002). Pairwise comparisons revealed that lane deviation increased from loop 1 to loop 2 (*p* = .042).

A significant interaction effect of day and time of day (*p* = .004) was found, showing that the time of day effect on lane deviation increased from one day to the next, as participants became more sleep deprived. No significant interaction effects were identified between day and time on task (*p* = .939), time of day and time on task (*p* = .602), or day, time of day, and time on task (*p* = .871).

#### Crashes

Significant main effects of day (*p* < .001), and time of day (*p* < .001; Figure 5b), but not time on task (*p* = .725; Figure 6b) were found for the number of crashes. Pairwise comparisons revealed a significant increase in crashes with sleep deprivation (*p* < .05), with more crashes near the circadian nadir (09:00) and less around the circadian peak (17:00).

A significant interaction effect was found between day and time of day (*p* = .001), demonstrating that the effect of time of day on crashes increased from one day to the next as participants became more sleep deprived. No significant interaction effects were identified between day and time on task (*p* = .912), time of day and time on task (*p* = .853), or day, time of day and time on task (*p* = .612).

#### Speed deviation

Significant main effects of day (*p* < .001), and time of day (*p* < .001; Figure 5c), but not time on task (*p* = .560; Figure 6c) were revealed for speed deviation. Pairwise comparisons revealed significant decreases in speed deviation between days 1 and 3, and days 2 and 3 (*p* < .001). A significant decrease in speed deviation was demonstrated from 09:00 to 17:00 (*p* < .001).

No significant interaction effects were found between day and time of day (*p* = .076), day and time on task (*p* = .610), time of day and time on task (*p* = .950), or day, time of day and time on task (*p* = .958).

#### Safe zone

Significant main effects of day (*p* < .001), and time of day (*p* < .001; Figure 5d), but not time on task (*p* = .148; Figure 6d) were identified for the time spent outside the safe zone. Pairwise comparisons revealed a significant increase in the time spent outside the safe zone as participants became more sleep deprived (*p* < .001). Reduced time was spent in the safe zone near the circadian nadir (09:00), whilst more time in the safe zone occurred near circadian peaks (17:00).

No significant interaction effects were revealed for day and time of day (*p* = .314), day and time on task (*p* = .835), time of day and time on task (*p* = .789), or day, time of day and time on task (*p* = .708).

### Measure of subjective workload—NASA-TLX

#### Overall score

**Figure 7.**
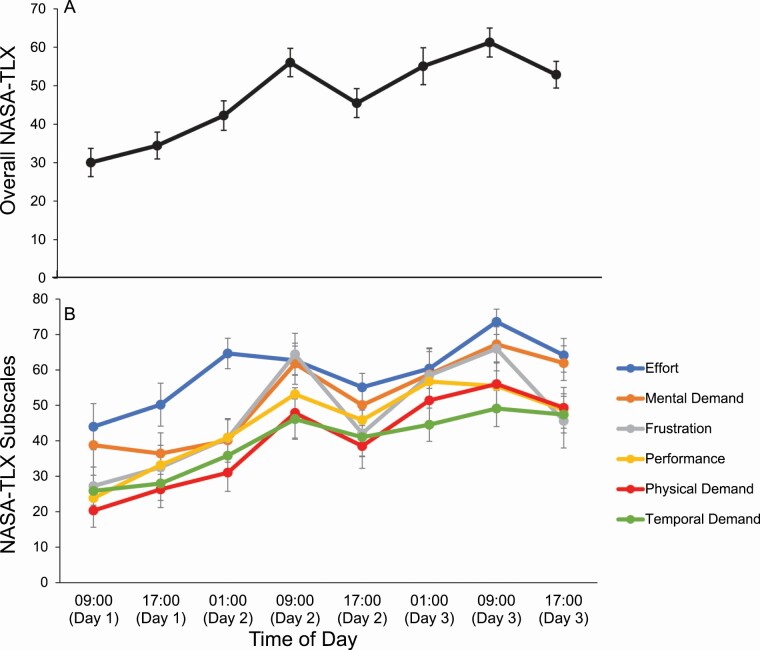
Mean NASA-Task Load Index (TLX) scores at each time of day. Mean scores across each time of day. Error bars display the standard error. The scale ranges from 0 (*very low*) to 100 (*very high*). (A) Overall NASA-TLX score. The overall score is the average of all the NASA-TLX subscales. (B) Average NASA-TLX subscales, targeting different workload components. The performance subscale was reverse scored to allow for clearer interpretations. Descriptive statistics are presented in table form in Table 3.

A significant main effect of day (*p* < .001), but not time of day (*p* = .078), were identified for the overall NASA-TLX score (Figure 7a). Pairwise comparisons revealed significant increases in subjective workload with increased sleep deprivation (*p* < .05). No significant interaction effect between day and time of day was found (*p* = .141).

#### Subscales

A significant main effect of day (*p* < .001), but not time of day were identified for the NASA-TLX subscales of mental demand, physical demand, temporal demand, performance, and effort (Table 4; Figure 7b). There were significant main effects of day and time of day for frustration (*p* < .001). Pairwsie comparisons revealed significant increases in mental demand and physical demand from one day to the next (*p* < .05; Table 3). Significant increases in temporal demand, performance, effort, and frustration were found from day to day (*p* < .05), except between days 2 and 3. Frustration scores significantly decreased from 09:00 to 17:00 (*p* = .047). There was no significant interaction effect between day and time of day for any of the subscales.

### Measure of behavioral and subjective alertness—PVT and KSS

**Figure 8.**
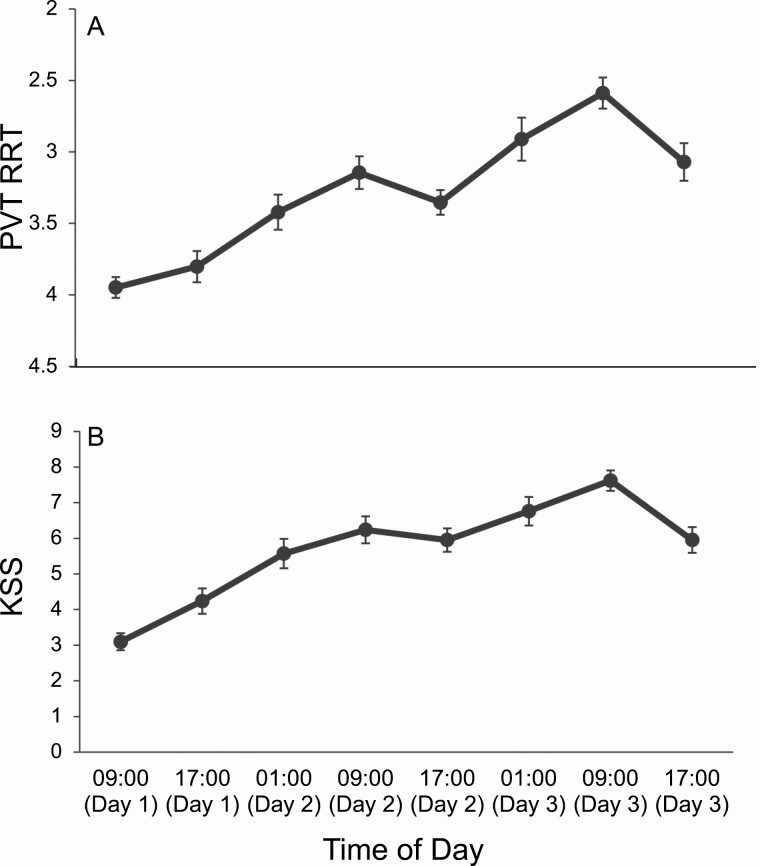
Mean scores at each time of day for Reciprocal Reaction Time (RRT) and Karolinska Sleepiness Scale (KSS). Mean scores across each time of day. Error bars display the standard error. (A) Reciprocal Reaction Time (RRT) for the 10-min Psychomotor Vigilance Task (PVT). The RRT is 1/reaction time × 1000. Higher scores indicate poorer performance. The y axis was flipped for clearer interpretations. (B) Self-reported Karolinska Sleepiness Scale (KSS) scores. Ratings range on a scale from 1 (*extremely alert*) to 9 (*extremely sleepy—fighting sleep*). Descriptive statistics are presented in table form in Table 3.

A significant main effect of day (*p* < .001), but not time of day (*p* = .073), were found for the PVT RRT (Figure 8a). Pairwise comparisons revealed that RRT significantly increased with increasing sleep deprivation (*p* < .001). There was not a significant day by time of day interaction effect for RRT (*p* = .063).

The KSS revealed a significant main effect of day (*p* < .001), but not time of day (*p* = .427), indicating that subjective alertness significantly decreased with sleep deprivation (*p* < .05; Figure 8b).

A significant day by time of day interaction effect (*p* < .001), was found, demonstrating that the effect of time of day on subjective alertness increased as participants became more sleep deprived.

## Discussion

This study investigated the combined effect of sleep deprivation, time of day, and workload on driving performance. All measures of driving performance, behavioral alertness, subjective alertness, and workload were impaired with increasing time awake. All driving measures and the frustration subscale in the NASA-TLX showed circadian variability, with worse performance in the early hours of the morning. Time on task, as a workload factor increased lane deviations.

Performance on all driving measures were significantly worse with sleep deprivation and in the early morning, consistent with previous research [4, 40–42]. Time on task, as a workload factor, also impacted driving performance as demonstrated by the significant increase in lane deviation from loop 1 to loop 2. In fact, there were performance decrements from 15-min into the driving task, specifically at 01:00. A previous study by Thiffault and Bergeron [19] found that lane deviation significantly increased in a 40-min monotonous afternoon driving task, with decrements beginning at 20-min. These time on task effects could be due to overworking of certain attentional neural circuits in the brain with longer time on task [13]. This causes these areas of the brain to essentially “fall asleep”. Hence, the longer time on task, the more performance decrements due to instability in these attention neural circuits. However, in the current study the same effects were not seen for crashes, speed deviation or safe zone. Lane deviation has been found previously to be sensitive in picking up changes in sleep deprivation [43]. But also, time on task as a workload factor may not result in consistently worse performance across all outcomes. It could have differential effects over time. This should be explored in future research. Nonetheless, this has significant implications when it comes to workers who are driving for extended periods, such as short and long-haul truck drivers. For instance, slight lane deviations can result in drifting into another lane and risk crashing with other road users.

All subjective measures, KSS, and NASA-TLX (except for effort and frustration), changed with sleep deprivation. Participants reported decreased alertness and increased workload with sleep deprivation. A recent study by Pesoli and colleagues [44] similarly identified an increase in NASA-TLX scores, however after only 24-h of sleep deprivation. In the current study both subjective scales were not administered at the circadian nadir for alertness, and this may explain why a significant effect of time of day was not found. The overall NASA-TLX score aligned with objective performance measures, as perceptions of workload demands were significantly higher at 09:00. It should be considered that participants had a relatively good introspection of their levels of alertness and sleepiness. This may be reflective of the acute sleep deprivation protocol employed in this study. Prior research has found that during chronic sleep deprivation individuals are not able to accurately assess their sleepiness and over time subjective ratings level out, while performance decrements continued [45]. From these findings, subjective measures could be implemented to ascertain the levels of workload and fatigue in individuals driving for extended periods of time. These easily accessible questionnaires could be administered prior and during a journey aiding countermeasure implementation, such as pulling over to take a break, consuming caffeine or taking a nap.

Time on task was the only workload factor explored in this study, however multiple interacting factors, including cognitive load, stress, and time pressure, contribute to workload [46]. Future research should consider exploring other workload factors. For example, cognitive load could be explored by comparing a high and low tempo driving task. This could be done by using the same track but adding extra stimuli to one, such as pedestrians and heavy traffic, to increase the cognitive load. Despite this, it is clear that time on task is an important workload factor for driving performance. Future research could employ the use of objective physiological workload measures, such as EEG to measure brain activity or eye tracking. Time on task was also only explored using two comparable blocks, rather than observing performance continuously across the duration of the task. This was not possible within the current study, due to the programing of the track, future research should consider exploring this.

Despite this driving task being sensitive to alterations in sustained attention [18, 19], the laboratory setting does not have the same stimulation and real-world pressures as on road driving, reducing its external validity. This should be considered when interpreting results. Nonetheless, the laboratory allows for control over extraneous variables such as caffeine consumption, light, and noise influences and makes replication easier due to standardized procedures.

Although, the sample consisted of young, healthy individuals, which does limit generalizability of the findings, research demonstrates shift workers who drive through the night are generally younger [47]. Future research should explore how well these findings are replicable within an older participant sample as they may have co-morbidities that could worsen results found here, such as a sleep disorder.

This study examined the importance of workload in combination with extended sleep deprivation and different times of the day on sustained attention driving performance. The potential impact that other workload factors may have on sustained attention should be investigated in future research. This study has implications for the wellbeing of long-haul driving populations, reinforcing the negative impact long monotonous driving has on driving performance and emphasizing the need to include workload in the messaging about accident risk to overall reduce the likelihood of motor accidents.

## Data Availability

The data underlying this article will be shared on reasonable request to the corresponding author.
